# Graft quality assessment during machine perfusion in liver transplantation: a review of current evidence and emerging strategies

**DOI:** 10.3389/frtra.2026.1892796

**Published:** 2026-07-28

**Authors:** Francisco Calderon Novoa, Franklin Gorospe, Nazia Selzner, Markus Selzner

**Affiliations:** 1 Multi-Organ Transplant Program, University Health Network/Toronto General Hospital, Toronto, ON, Canada; 2 Perioperative Services, University Health Network, Faculty of Community Services, Toronto Metropolitan University, Toronto, ON, Canada; 3 Department of Medicine, University of Toronto, Toronto, ON, Canada

**Keywords:** hypothermic machine perfusion (HMP), liver transplantation, machine perfusion (MP), normothermic machine perfusion (NMP), organ preservation, viability assessment

## Abstract

Machine perfusion (MP) has transformed liver transplantation by enabling organ reconditioning, extending preservation times, and providing a dynamic platform for graft quality assessment. The ability to evaluate organ viability represents one of the most clinically impactful applications of this technology. As different perfusion modalities develop, distinct strategies have emerged to characterize graft viability. However, despite the growing evidence, significant heterogeneity remains in the parameters and thresholds employed, varying between each MP modality, as well as between centers. This review provides a comprehensive and critical synthesis of available evidence on viability assessment during each perfusion modality, and discusses promising new strategies in the race to the holy grail of viability assessment: prevention of both primary non-function (PNF) as well as the prediction of ischemic cholangiopathy (IC), so as to achieve tangible benefits in patient-centered outcomes.

## Introduction

Organ shortages remain the defining constraint of liver transplantation, driving centers worldwide toward increasingly liberal use of extended criteria donors (ECDs) (1, 2). While this strategy expands the donor pool, it carries an increased risk of primary non-function (PNF) and early allograft dysfunction (EAD), both of which bear significant clinical consequences, including acute kidney injury, elevated perioperative mortality, and reduced long-term patient survival (3–5). Conventional risk stratification tools such as the Liver Donor Risk Index (LDRI) (6), the UK Donation after circulatory death (DCD) Risk Score (7), and the Balance of Risk (BAR) system (8) provide pre-procurement risk estimates but have shown imperfect correlation with post-transplant outcomes across different publications (9, 10), leaving an unbridged gap between pretransplant assessment and post-transplant graft performance.

Machine perfusion (MP) has emerged as the most promising answer to this challenge. With close to 20 years of evidence now supporting its benefits in organ reconditioning, extending preservation times, and improving postoperative outcomes, MP offers a unique opportunity to evaluate grafts under controlled conditions before implantation (11). Among the available technologies, normothermic machine perfusion (NMP) has classically been described as the one that most closely captures physiological conditions and has been positioned as the gold standard for quality assessment (12, 13). However, viability assessment is not exclusive to NMP, and emerging evidence suggests that hypothermic machine perfusion (HMP) and normothermic regional perfusion (NRP) also provide actionable information about graft function, and that each modality carries distinct advantages and limitations in this context.

This review focuses specifically on the evidence for graft quality assessment across all three MP modalities, examines classical and emerging biomarkers that may enhance decision-making, and discusses unresolved challenges, including the persistent lack of standardized criteria and the evolving definition of “viability” itself. The role of MP as a preservation strategy has already been established and is beyond the scope of this article. Rather, this will encompass the available options for viability assessment in each of the three currently employed MP strategies (HMP, NMP, and NRP), as well as promising updates. Of note, while HMP encompasses both anoxic and oxygenated variants, the available clinical evidence on viability assessment in the hypothermic setting derives almost exclusively from oxygenated modalities, the review of HMP assessment therefore focuses on these techniques.

## Viability assessment during hypothermic machine perfusion

HMP encompasses a spectrum of approaches that vary in temperature (4–10 °C), oxygenation (anoxic vs. oxygenated), and circuit configuration (continuous vs. pulsatile). The most used variants in the literature include anoxic HMP, portal-only hypothermic oxygenated perfusion (HOPE), and dual hypothermic oxygenated perfusion (D-HOPE). While clinical evidence consistently demonstrates that these techniques reduce ischemia-reperfusion injury and improve outcomes relative to static cold storage (SCS), their utility as platforms for quality assessment has always been challenged. Arrhenius kinetics dictate a drop of 50% of the metabolic rate with every 10 °C drop (14) and have been used as the cornerstone argument for organ preservation using static cold storage. This metabolic suppression limits the range of evaluable functions, but does not eliminate assessment opportunities entirely, as shown below.

### Biochemical markers in the perfusate

The first clinical application of liver HMP by Guarrera et al. included an exploratory analysis of perfusate injury markers. It detected correlations between aspartate aminotransferase (AST), alanine aminotransferase (ALT), and lactate dehydrogenase (LDH) levels at two hours and postoperative transaminase patterns (15). Following these observations, studies have dealt with mixed results over the last fifteen years. Rayar et al. identified perfusate AST and ALT cutoffs of ≥800 U/L as predictors of EAD in a cohort of 25 extended criteria donation after brain death (DBD) livers undergoing HOPE, with area under the curve (AUC) values of 0.92 and 0.91, respectively (16). In contrast, Patrono et al. found no independent association between perfusate biochemical markers and EAD in 57 D-HOPE-treated livers after weight normalization (17), and Muller et al. reported similarly disappointing results in 106 high-risk livers: perfusate AST and ALT showed only moderate predictive accuracy for 90-day graft survival (AUC 0.747 and 0.811, respectively) and were poor surrogates for severe EAD and graft loss (18). A recent study identified lactate levels as adequate predictors of postoperative EAD, with discriminative capability increasing at longer perfusion times (AUC 0.841, 95% CI 0.683–0.999, *p* = 0.009) in a cohort of 26 livers undergoing D-HOPE before transplantation (19). Further analysis showed correlation with post-transplant peak transaminase levels and extended hospital stays.

Acknowledging the limitations of HMP perfusate injury markers, Patrono et al. explored microdialysis (MD) as an alternative real-time sampling modality (20), demonstrating diverging kinetic profiles of lactate and glucose in MD samples between EAD and non-EAD livers at 2 h of HOPE, with significantly higher levels in those that subsequently developed dysfunction. These differences were not mirrored by perfusate levels, suggesting that interstitial fluid dynamics may capture metabolic signals not accessible through circuit-based analysis. While intriguing, this approach requires further validation in larger prospective cohorts.

Taken together, published data do not support a robust role for classical injury markers as reliable viability indicators during liver HMP. The emerging consensus leans toward these markers being informative at best and misleading at worst, and caution is warranted in their application to clinical decision-making.

### Flavin mononucleotide: a mitochondrial injury biomarker

FMN has emerged as one of the most promising biomarkers in the HMP literature. This flavin moiety is released from mitochondrial complex I upon cellular injury (21) and has been linked to ischemic damage levels in brain ischemia-reperfusion models (22–24). Initial characterization by Muller et al. in 61 HOPE-treated livers demonstrated that perfusate FMN levels correlated strongly with coagulation factor levels and peak post-transplant transaminases, associations that perfusate AST, ALT, or LDH levels were unable to capture (18). Subsequent mechanistic work by the Zurich group linked HOPE-mediated mitochondrial conditioning to reduced succinate-driven reactive oxygen species production, placing FMN as a mechanistic marker and a surrogate for mitochondrial damage (25).

A large multicenter study by Eden et al. analyzed perfusate samples from 473 livers (315 DCD, 158 DBD) (26). FMN demonstrated high discriminative accuracy for graft loss due to PNF or cholangiopathy (c-statistic 0.77; 95% CI 0.70–0.85) and for non-anastomotic strictures and the need for renal replacement therapy (c-statistic 0.76; 95% CI 0.71–0.81). This study was the first to propose a risk stratification framework for livers on HMP based on FMN thresholds: low (<5,165 A.U.), intermediate (5,165–6,415 A.U.), and high risk (>6,415 A.U.). External validation by Dingfelder et al. in 57 HOPE-treated livers confirmed FMN predictive utility for EAD (optimal cutoff 10.6 ng/mL; sensitivity 86.7%, specificity 62.5%) and, notably, for 1-year patient survival (27). Most recently, an internationally validated quantification protocol has been published to standardize FMN measurement across centers (28). Increasing data from other centers is starting to build up, associating higher FMN levels during HMP with increased EAD rates (c-statistic 0.833, 95% CI 0.569–1.000, *p* = 0.016) (19). Additional studies by the Groningen group have shown a clear association between HOPE FMN levels and acceptance of grafts during NMP: grafts accepted for transplantation (67% of the cohort, *n* = 78) had significantly lower FMN levels across different timepoints (30, 60, and 120 min) compared to those rejected (29). This study on 103 extended criteria DCD livers calculated viability thresholds that adequately correlate to the Groningen criteria and could potentially alleviate the burden of resources for both livers that can be implanted directly and those that can be discarded without further investigation. While appealing, this concept tends to minimize the role of NMP to merely an assessment tool, without considering the potential benefits and protective effects of NMP following HOPE. NMP has demonstrated the capacity to recondition grafts by restoring cellular function at physiological temperature. Additionally, the Groningen protocol which includes HMP, controlled oxygenated rewarming (COR) and NMP: it is likely that this protocol allows for a slow recovery of grafts with subtle reoxygenation initially, which in term reduces the succinate upon reperfusion, minimizing IRI. By allowing for slow rewarming, the graft benefits from the slow infusion of increasing levels of oxygen; that limits the amount of succinate generated until all the cellular function is restored. By ignoring the warm phase of this protocol, there would be a missed window of opportunity for active ex-vivo reconditioning and repair, as well as potential therapeutic interventions.

The specificity of FMN to the liver and perfusion modality should not be overlooked. Attempts to replicate findings in kidney transplantation have yielded conflicting results: a pilot study found associations between perfusate FMN and delayed graft function (DGF) (30), while a larger follow-up study found no significant correlation with postoperative outcomes including creatinine, immediate graft function, or 1-year rejection (31). This suggests that FMN may be an organ-specific or, more likely, a modality-specific biomarker, and a “one-size-fits-all” approach may not be sufficient for its clinical implementation.

#### Hemodynamic Parameters

Experience with hepatic hemodynamics during HMP is largely derived from preclinical models and small clinical series. Liu et al. demonstrated in a porcine model that hepatic artery vascular resistance during HMP varied according to increasing ischemic times and proposed a composite damage index based on five HMP variables (32). Clinical data, however, remain scant. Monbaliu et al. compared non-transplantable with potentially transplantable unallocated livers undergoing HOPE and found that despite significant differences in AST and LDH levels, arterial and portal resistances were virtually identical between groups (33). Patrono et al. observed that EAD-free livers exhibited steeper drops in arterial resistance during HOPE, though both arterial and portal resistance correlated poorly with peak postoperative transaminases (34). This lack of consistency across series suggests that hemodynamic parameters should not be used as stand-alone viability criteria during liver HMP, and their use should be interpreted alongside biochemical data, if at all.

## Viability assessment during normothermic machine perfusion

NMP maintains grafts at physiological temperature (36–38 °C) with oxygenated, blood-based perfusate, enabling near-complete restoration of cellular metabolism (35). This normothermic environment is uniquely suited to organ quality assessment: the organ is isolated from systemic confounders, and multiple variables involving hepatocellular, cholangiocyte, and endothelial functions can be closely monitored. The concept of viability assessment is therefore the foundational cornerstone of liver NMP and has been the primary focus of research throughout the technology's clinical development.

### Development of core viability criteria

The foundations for NMP viability assessment were established in a landmark porcine study by the Oxford group, where variable warm ischemia time (WIT) and preservation duration directly influenced transplant outcomes, and candidate markers (bile output, lactate, AST, ALT, hyaluronic acid, and hemodynamic parameters) were identified as predictors of successful transplantation (36). The first human liver NMP series, reported by Ravikumar et al. in 2016, established safety and feasibility, with criteria based on hemodynamic stability, pH normalization, and bile production (37). Following these foundational criteria, multiple centers have established and published their own frameworks.

The “Birmingham Criteria,” arising from assessment of initially declined livers placed on NMP, required specific thresholds after 3 h of perfusion (38): lactate ≤ 2.5 mmol/L or active bile production, plus at least two of: (A) perfusate pH ≥ 7.30; (B) hepatic artery flow ≥150 mL/min and portal vein flow ≥500 mL/min; (C) homogeneous macroscopic graft appearance. Four of five livers meeting these criteria were successfully transplanted with 100% 6-month survival. These criteria were subsequently applied and expanded in the VITTAL trial, which incorporated glucose metabolism and achieved a liver rescue rate of 71%, with 100% survival at 3 months and 83% graft survival at 1 year in 22 of 31 perfused livers transplanted (39, 40). Notably, 5-year follow-up data revealed considerable late attrition from IC with graft loss due exclusively to this cause, highlighting the limitations of lactate-based criteria in predicting biliary complications and the importance of bile assessment (40).

The Toronto group contributed initial experience with Steen solution as a cell-free perfusate alternative to albumin, with graft acceptance requiring homogeneous perfusion, hepatic artery flow ≥150 mL/min, portal vein flow ≥800 mL/min, lactate ≤2.5 mmol/L, and active bile production at four hours, showing short-term outcomes comparable to those from SCS livers (41). The Cambridge group reported outcomes on 48 perfused livers, with one of the highest recorded discard rates (53%). However, this allowed for characterization of underperforming grafts by bile pH and glucose dynamics, perfusate pH instability, glucose trends, peak lactate fall rate, and ALT <6,000 IU/L at 2 h (42). While cutoffs and timepoints differ, most centers heavily rely on lactate clearance as one of the main indicators.

### Variability across centers

Despite broad convergence on core parameters (hemodynamic flows, lactate clearance, pH, bile production, and macroscopic appearance), substantial variability persists in cutoff values, assessed timepoints, and supplementary criteria. A meta-analysis of 16 studies encompassing 568 livers identified mean acceptance rates of 91% for DBD and 74% for DCD livers (43). When more stringent criteria were applied, particularly cholangiocyte-specific parameters and earlier lactate assessment, discard rates were up to 20%–22% higher in DCD vs. DBD cohorts. Of note, vascular flows were included as viability criteria in most studies but showed no statistically significant difference between accepted and discarded livers in this analysis, questioning their role as independent viability criteria.

These findings prompt a fundamental question: What does viability mean in 2025? Early NMP trials were primarily designed to prevent PNF, and this goal has been largely achieved, as shown by PNF rates of 0% for DBD and 1.6% for DCD livers in the meta-analysis above. However, DCD IC rates were 12% across all studies. This correlates with the tolerance of different cell populations to ischemia and reperfusion. Studies have shown that while bile duct epithelial cells are more resistant to lethal anoxic injury than hepatocytes, the latter suffer greater injury upon reperfusion (44). When considering that the main functions of cholangiocytes differ from those of hepatocytes such as lactate clearance, it is unsurprising that current viability criteria that focus on hepatocellular markers fail to capture the ongoing damage on biliary epithelia. While this number is lower than that reported for SCS-DCD transplantation, it still suggests a limit in NMP's capacity to “treat” grafts, hinting towards positive selection of healthy livers as the main driver. The contemporary focus has accordingly shifted toward IC, biliary complications, and longer-term graft function as targets for viability assessment.

### Cholangiocellular criteria and bile quality

Given the high rates of IC in DCD transplantation and its devastating impact on graft survival, cholangiocellular assessment has become an increasingly prominent component of viability frameworks. The physiological basis rests on the biliary epithelium's active transport functions: healthy cholangiocytes produce alkaline, low-glucose bile through bicarbonate secretion and glucose reabsorption (45). Matton et al. characterized bile composition during NMP, demonstrating that injured biliary epithelia failed to alkalinize bile and exhibited elevated glucose levels (46). Optimal thresholds identified were: bicarbonate ≥18 mmol/L, pH >7.48, glucose <16 mmol/L, and bile/perfusate glucose ratio <0.67. A follow-up study from the Groningen group confirmed that bile glucose and bicarbonate are more stable parameters than pH, which can drift if collection technique is inconsistent (47).

The Groningen group has extended these findings through sequential HMP-COR-NMP protocols, demonstrating that combined perfusion approaches can enable transplantation of high-risk DCD livers with outcomes comparable to DBD grafts (48). Incorporation of bile quality assessment into “traffic light” viability systems, which assign red, yellow, and green zones across multiple parameters, is gaining wider adoption and allows dynamic reassessment of borderline grafts over extended perfusion windows rather than application of binary pass/fail criteria (48).

### Impact of NMP on patient outcomes

Although viability assessment may seem purely academic, it is important to recognize that the ultimate measure of successful viability criteria must derive from patient-centered results. In the United States alone, approximately 1 in 4 patients listed for liver transplantation either dies on the waitlist or becomes too sick to undergo transplantation before an organ becomes available (49). Every viable graft incorrectly discarded represents a missed transplant opportunity for one of these patients. MP has already demonstrated its capacity to reduce organ discard rates at a population level, with NMP identified as the only preservation modality independently associated with reduced graft non-utilization in large registry analyses (50). Center-level data confirm that implementation of NMP-based programs is associated with significant reductions in waitlist time and pretransplant mortality (51, 52). A comprehensive single-center analysis of over 1,000 liver transplants comparing 342 NMP-preserved livers with 744 SCS-preserved livers (53) showed that NMP reduced EAD. Importantly, NMP reduced hospital length of stay and ICU length of stay in both DCD and DBD transplantation, translating into patients spending less time in hospital and more time at home. In the same line, NMP was able to reduce the risk of 1-year readmission by 86% for DCD transplant and 53% in DBD transplantation, with only 10% of DCD-NMP cases being readmitted over 1 year. This highlights the potential of NMP to improve patient outcomes and quality of life.

These gains, however, are only as valid as the quality of the viability criteria. An assessment framework that is too permissive risks transplanting non-viable grafts with consequent PNF and biliary complications; one that is too restrictive perpetuates unnecessary discard and condemns patients to longer waitlist exposure. Viability assessment has therefore become a key determinant of patient outcomes.

## Viability assessment during normothermic regional perfusion

NRP represents a fundamentally different approach to MP: rather than ex-vivo organ evaluation, it applies normothermic oxygenated perfusion *in situ* following circulatory death, prior to organ retrieval. By re-establishing metabolic activity in the donor before procurement, NRP enables organ reconditioning while also serving as a preliminary quality assessment. The technique has been applied in both controlled DCD (cDCD) and uncontrolled DCD (uDCD) settings, with abdominal (A-NRP) and thoracoabdominal (TA-NRP) configurations. Briefly, A-NRP encompasses the perfusion of abdominal organs only. By cannulation of the infrarenal aorta or femoral artery and inferior vena cava followed by either thoracic or abdominal clamp of the descending aorta, perfusion is contained within the abdominal compartment (54). In TA-NRP, there is no diaphragmatic clamp, and perfusion extends to include the thoracic cavity, reperfusing both abdominal and thoracic organs simultaneously (55).

### Evolution of NRP viability criteria

Early NRP programs lacked formalized viability criteria, and rejection of grafts was largely guided by macroscopic assessment and non-standardized biochemical thresholds. The Barcelona group's initial experience, labeled normothermic extracorporeal membrane oxygenation (NECMO), relied heavily on macroscopic steatosis estimation and AST/ALT elevation upon perfusion initiation as rejection criteria in uDCD donors (56). Hessheimer et al.'s multicenter comparison of NRP vs. super-rapid recovery (SRR) in cDCD incorporated AST/ALT thresholds of ≤3 times the upper limit of normal (ULN) at circuit initiation and ≤ 4 times the ULN at completion. However, macroscopic appearance remained the leading cause of discard (57).

More structured criteria emerged from the French and Italian experiences. Antoine et al. applied stringent criteria in 152 cDCD livers, requiring AST/ALT < 4x ULN in at least 3 consecutive samples and mandatory liver biopsy excluding steatosis >20% or fibrosis ≥F2. Following these criteria, 1-year survival reached 95% and only one ITBL case was observed (58). The Milan group (De Carlis et al.) applied similar criteria in a sequential NRP + HOPE cohort: livers with ALT >1,000 IU/L, rising lactate after four hours, suboptimal macroscopic appearance, steatosis ≥30%, or fibrosis ≥F2 would be rejected, with acceptance rates of 86.5% for cDCD and 73.1% for uDCD. However, PNF rates of 5% remained a concern in this cohort (59).

### Lactate clearance as a Key biomarker

Lactate dynamics have attracted particular interest as a viability marker. Ghinolfi et al. observed lower final lactate levels in accepted vs. rejected livers in a cDCD cohort of 34 livers undergoing NRP before either D-HOPE or NMP (60). A larger multicenter study by the same group demonstrated that perfusate lactate levels during the first 120 min of NRP directly correlated with early allograft function, post-reperfusion syndrome (PRS), and 90-day graft loss, with a lactate reduction of 26.4% over two hours identified as the optimal threshold (61). Comparable findings were reported by Steinberg et al., where lactate reduction and lactate-normalized oxygen delivery were the strongest predictors of graft acceptance in 27 NRP-treated livers (62). However, lactate clearance during NRP has important caveats that are frequently underappreciated. In A-NRP, a diaphragmatic cross-clamp isolates the abdominal compartment, limiting recirculation of blood within a relatively constrained volume. In TA-NRP, by contrast, the entire thoracoabdominal territory including the heart and lungs is perfused, dramatically increasing the volume of ischemic tissue beds contributing lactate to the circuit. This difference is clear when comparing the requirements for both priming volumes, as well as acceptable flow ranges between A-NRP and TA-NRP. This leads to differences in absolute lactate levels starting levels as well as clearance kinetics, making direct comparison across configurations challenging, and reference ranges established in A-NRP cannot be uncritically applied to TA-NRP (63). While this does not invalidate lactate as a biomarker, it underscores the need for standardized perfusate handling and reference ranges that account for circuit composition.

### Limitations of viability assessment in NRP

Viability assessment during NRP faces several structural challenges. In contrast to NMP, NRP assessment occurs within a complex circulatory circuit influenced by bowel ischemia, variable WIT, and hemodynamic instability. This results in a higher proportion of viability decisions being driven by subjective criteria: macroscopic appearance accounts for approximately 50% of all NRP discards in recent grouped analyses (43). Major transplantation societies, including the European and American, have published guidelines endorsing NRP and recommending assessment of stable flows, declining lactate, AST levels, and satisfactory macroscopic appearance, but without outline of hard cutoffs (79–83). Given the lack of solid evidence for viability assessment during NRP, experts advocate for sequential NMP following NRP for those organs deemed “suboptimal” prior to discard (43). While costly, this strategy may help in rescuing livers in the twilight zone of viability.

### The role of combining perfusion modalities in viability assessment

Combined perfusion (i.e., NRP + HMP/NMP, HMP + NMP) is an attractive strategy in several ways. To begin, it has the potential for enhancing outcomes by adding the cumulative effects of each mechanism. The Birmingham group's early work allocated 10 discarded human livers to either 6 h of NMP (*n* = 5) or 2 h of HMP followed by 2 h of NMP with a solution using Hemopure as the oxygen carrier (*n* = 5) and assessed both groups according to the viability criteria described above (64). Analysis revealed a lower expression of oxidative stress markers such as 4-hydroxinonenal or CD14 expression, as well as inflammation markers (CD11b, VCAM-1) in combined perfusion livers compared to NMP alone. While a small cohort, all livers in the combined perfusion group met all viability criteria, while only 2 of those in the NMP did. This proof-of-concept study showed that combined perfusion was not only feasible, but that it yielded the benefits of both modalities: Mitochondrial repair and succinate metabolism during the cold to mitigate NMP-induced IRI along with viability assessment during NMP. It is likely that use of open system perfusion modalities along with acellular perfusates that can be used both in the cold and the warm make combined perfusion simpler, easier, more accessible, and cheaper, optimizing overall results. While this study bears great insight on the potential benefits of combining the two ex-situ perfusion modalities, there are still no prospective trials comparing combined methods to either method alone.

There have been several studies exploring combined *in-situ* and ex-situ perfusion. Ghinolfi et al. published their experience with 20 grafts (*n* = 11 uDCDs, *n* = 9 cDCDs) considered

eligible for LT. All were procured with *in-situ* NRP, followed by NMP (*n* = 9) or DHOPE (*n* = 11) (60). This strategy yielded a 90% transplant rate, with 18/20 livers eventually transplanted. In 2021, De Carlis et al. compared NRP + DHOPE (*n* = 37) with NRP + SCS (*n* = 37) from another European center's cohort (59). Viability assessment was not the focus of this study, but rather it showed that combined NRP + DHOPE allowed for the use of livers with longer WIT and higher DCD-Risk scores with comparable PNF and ITBS rates, along with reduced AKI rates and severity. While not significant, 2-year graft and patient survival also showed enhanced results when combining perfusion techniques. A 2021 study compared DCD grafts from different European centers: group A consisted of grafts undergoing NRP + HOPE, or NRP + SCS. While group A encompassed Italian data and had longer no-flow, total and functional WIT than group B (30.5 ± 7.7 vs. 20.5 ± 4.1; 56.5 ± 20.4 vs. 39.1 ± 21.6; 41.9 ± 12.5 vs. 25.5 ± 3.7; respectively, *p* < .05), there were similar rates of EAD, PNF, IC, non-IC biliary complications and re-transplantation. The main highlight of this small (*n* = 19 and *n* = 17, respectively) study was that the cohorts presented similar postoperative outcomes while using very different DCD risk profiles: over 60% of the Italian cohort (group A) had UKDCD risk scores of 11 or higher, while only 17.6% in the French cohort. While this is likely driven by the mandatory 20-minute no touch period in Italy during the time this study was conducted, it showed that NRP combined with HOPE could safely expand the limits of DCD transplantation. Viability was determined by each country's protocol, and included biopsy in both cohorts, as well as macroscopic assessment, target flows and lactate and ALT trends throughout NRP. In this study, viability assessment was performed only during NRP. This is unsurprising, given that viability is still not routinely performed during HOPE.

There are some more studies that look at outcomes following NRP + NMP. Croome et al. (65) showed that either NRP alone or NRP + NMP enhanced outcomes of DCD donation when compared to SCS, with no cases of IC in either cohort. In this series, there were three circumstances in which the authors advocated for sequential perfusion: long travel times or logistical issues, complicated recipients and need for further assessment of “marginal” cases. In this last scenario, authors decided to place livers that had terminal lactate levels after NRP that were either stagnant or increased throughout perfusion. Additional reasons for further assessment in this series included 3 cases of prolonged functional WIT, and 1 case with elevated AST/ALT during NRP. All these cases were assessed during NRP and successfully transplanted. While the authors make a point in highlighting that most of, if not all, of these livers would have been discarded without machine perfusion, there is no data on accepted/rejected rates to determine the actual value of combined NRP + NMP in this study.

While dedicated studies looking into the role of sequential perfusion as a method to assess and “rescue” marginal livers are scarce, this approach, in particular combining NRP with NMP has great potential for assessing livers and expanding the available grafts.

## Emerging strategies in graft quality assessment

The limitations of conventional viability parameters have motivated a broad search for complementary biomarkers and real-time assessment tools. Synthetic function is surprisingly absent from conventional NMP viability criteria, despite representing one of the liver's most relevant functions. Eshmuminov et al. demonstrated that livers preserved on NMP for up to one week progressively restored coagulation factor synthesis, including factor V, factor VII, fibrinogen, and antithrombin, with near physiological levels within the first two days (66). Van den Boom et al. extended this approach by investigating the international normalized ratio (INR) as a point-of-care coagulation marker during NMP (67). After applying correction factors for the highly heparinized perfusate, decreasing INR trends were identified in 17 of 18 perfused livers at 5 h. Larger prospective studies are required before coagulation-based criteria can be formally recommended.

Downstream cytokine levels have also been of interest. Pezzati et al. analyzed perfusate cytokine levels in 30 grafts from donors over 79 years old undergoing NMP, identifying positive correlations between IL-6 and postoperative bilirubin levels, IL-10 and ICU stay, and TNF-alpha and INR levels. Most importantly, IL-6 levels at 2 h correlated directly with graft failure (*p* = 0.02) (68). While intriguing, cytokine assessment remains time-intensive, limiting current clinical applicability.

Cell-free microRNAs, small non-coding RNAs released during cellular injury, have been studied as markers in MP perfusate. Matton et al. demonstrated strong correlations between perfusate microRNA levels and peak AST and biliary LDH following 6 h of NMP in 12 discarded donor livers, profiling microRNAs as potential predictors of EAD, PNF, and graft loss (69). The primary limitation is processing time: 4–5 h may be incompatible with real-time decision-making in current workflows. Graft-derived cell-free DNA (gdcfDNA) has also shown promise in a proof-of-concept study of 10 NMP-perfused livers, where perfusate and bile levels correlated with graft acceptance and discard status (70). Beyond injury markers, direct assessment of functional hepatic capacity using the LiMAx test and mitochondrial respirometry has shown early promise, with proof-of-concept and clinical studies respectively demonstrating feasibility and correlation with outcomes (70, 71). Raman spectroscopy has similarly been evaluated for real-time monitoring of mitochondrial oxygenation (72).

Dynamic imaging modalities offer real-time graft evaluation as an adjunct to current assessments. Indocyanine green (ICG) fluorescence has been evaluated as a surrogate for hepatic clearance during NMP. In a porcine model, Goto et al. demonstrated distinct fluorescent profiles across varying ischemic conditions (73). The first standardized human methodology for ICG clearance assessment during NMP recently showed higher clearance in accepted vs. discarded grafts and correlation with L-GRAFT scores at 1 week (74). Hyperspectral imaging (HSI), a technology capturing tissue oxygen saturation (StO2), near-infrared (NIR) perfusion index, and tissue water index (TWI), has been evaluated in two NMP cohorts. Fodor et al. reported progressive increases in StO2, NIR index, and TWI in 21 perfused livers, with higher hemoglobin concentrations in discarded grafts and correlation with lactate levels (75). Kneifel et al. confirmed StO2 and tissue hemoglobin index accurately predicted lactate levels at 1, 2, and 4 h in 25 livers, and a subsequent follow-up identified lower NIR index values in livers that later developed EAD in a cohort of 37 cases (76, 84).

As in most realms within medicine, artificial intelligence (AI) is gaining momentum within organ transplantation. While the most obvious use for AI lies optimization of organ allocation and waitlist mortality. Bertisimas et al. developed the optimized prediction of mortality (OPOM), which when tested against the conventional MELD score vastly outperformed the later, reducing mortality by 418 per every year analyzed across all demographics, regions and candidates, allowing for more equitable organ allocation (77). Another study looked at over 105,000 patients listed in the U.S. over 20 years (78). After splitting the sample in 60:20:20 between training, validation and testing, the neural-network 90-day waitlist mortality prediction rate was more sensitive than MELD-based models. Given the capacity to use machine learning (ML) to collate multiple variables between both donor and recipient and across multiple potential donor-recipient matches to further optimize organ utilization and patient outcomes, it is very likely that AI holds multiple benefits within the MP space. While this field remains nascent and current datasets remain small, it is clear that AI-assisted decision support tools must grow with the growing data on perfusion across the world, having the potential to synthesize multiparameter inputs into real-time viability scores, reducing subjectivity and improving consistency across institutions. Ultimately, the goal of these assessment strategies is patient-centered: a graft that is correctly identified as viable results in a successful transplant, a shorter waiting time, and potentially a life saved. Conversely, unnecessary discard of a viable graft represents a missed opportunity for a patient who may deteriorate or die on the waitlist (85).

## Discussion

The field of viability assessment in MP has made substantial progress over the past decade, but several challenges remain. No universal criteria exist across any perfusion modality, and the diversity of parameters, thresholds, and timepoints reflects both practice variation and genuine clinical uncertainty. The use of different perfusion modalities, circuits, and acceptance criteria between centers makes consistency a challenge. Systematic reviews confirm that variable stringency directly affects acceptance rates, and that some commonly used parameters such as vascular flows provide limited discriminatory value (43).

The definition of viability is also evolving. The early NMP literature was almost entirely focused on preventing PNF. As PNF rates have fallen to near zero in experienced centers, the goalposts have shifted toward IC: a more nuanced complication that develops over weeks to months and is poorly predicted by current hepatocellular criteria. Cholangiocellular assessment, as pioneered by the Groningen group, represents the most direct response to this challenge. However, biliary quality data require technical standardization, and current biochemical parameters alone may fail to entirely predict IC, as seen in the VITTAL trial.

The emerging consensus favors dynamic over static assessment. The transition from fixed-timepoint, threshold-based decisions to “traffic light” systems that allow extended perfusion and serial reassessment reflects growing recognition that single-timepoint snapshots are insufficient for complex grafts. Novel biomarkers, particularly FMN, are moving from proof-of-concept toward early clinical validation with an internationally validated quantification protocol now available. Before FMN can be incorporated into routine clinical decision-making, however, prospective multicenter trials with standardized measurement protocols and pre-specified outcome endpoints are needed to confirm its predictive thresholds across different donor populations, perfusion circuits, modalities, and perfusate compositions. The economic implications of FMN-guided strategies, including the potential to shorten or bypass NMP in low-risk grafts, must also be formally assessed against the cost of missed reconditioning opportunities or incorrectly discarded organs. Additionally, the cost of not only equipment, materials and reagents necessary for FMN testing, but also the availability and cost of qualified professionals to perform laboratory grade assays must be factored in. Imaging modalities such as HSI offer an attractive combination of continuous, non-invasive monitoring and multiparameter output, but require larger prospective studies to establish clinical thresholds. None of these approaches are likely to replace conventional criteria in isolation; rather, they are best conceived as complementary dimensions in a multi-domain assessment framework. Finally, NRP assessment deserves particular attention as a research priority, as its assessment infrastructure lags behind NMP, and confounding effects of non-hepatic ischemia on perfusate markers remain a fundamental challenge.

## Conclusion

MP has irrevocably changed the trajectory of liver transplantation. Its capacity for graft quality assessment across modalities is one of its most valuable and clinically actionable dimensions. While NMP currently remains the modality best suited for comprehensive viability evaluation, HOPE provides meaningful prognostic information through FMN, and NRP offers preliminary *in-situ* assessment prior to retrieval. The current landscape is characterized by expanding evidence, increasing center-specific experience, and a shifting focus from PNF prevention toward IC prediction and long-term graft quality. The integration of emerging biomarkers, dynamic assessment protocols, and imaging technologies into standardized, multiparameter frameworks is the logical next step, requiring prospective multicenter collaboration. As the field matures, so too must the definition of viability: just as we consider benchmarks for patient and graft survival at 1 and 5 years, the criteria used must adjust accordingly @.
